# Lithiation of white button mushrooms (*Agaricus bisporus*) using lithium-fortified substrate: effect of fortification levels on Li uptake and on other trace elements

**DOI:** 10.1007/s11356-021-13984-6

**Published:** 2021-04-30

**Authors:** Sviatlana Pankavec, Jerzy Falandysz, Izabela Komorowicz, Anetta Hanć, Danuta Barałkiewicz, Alwyn R. Fernandes

**Affiliations:** 1 Environmental Chemistry and Ecotoxicology, University of Gdańsk, 63 Wita Stwosza Str, 80-308 Gdańsk, Poland; 2 Environmental and Computational Chemistry Group, School of Pharmaceutical Sciences, University of Cartagena, Zaragocilla Campus, 130015 Cartagena, Colombia; 3 Department of Trace Elements Analysis by Spectroscopy Methods, Faculty of Chemistry, Adam Mickiewicz University in Poznań, 89b Umultowska Street, 61-614 Poznań, Poland; 4 School of Environmental Sciences, University of East Anglia, Norwich, NR4 7TJ UK

**Keywords:** Bio-fortification, Food, Food safety, Medicine, Mushrooms, Trace elements

## Abstract

High doses of lithium salts are used for the treatment or prevention of episodes of mania in bipolar disorder, but the medication is rapidly excreted and also shows side effects. Li may also be beneficial in people with mood disorders. Nutritionally, popular foods such as wild and cultivated mushrooms have low Li contents. This study evaluated the Li enrichment of white *Agaricus bisporus* mushrooms using Li_2_CO_3_ solutions to fortify the commercial growing substrate at various concentrations from 1.0 to 500 mg kg^−1^ dry weight (dw). Fortification of up to 100 mg kg^−1^ dw resulted in a significant (*p* < 0.01) dose-dependent increase in the accumulation of Li in mushroom, but the highest fortification level was found to be detrimental to fruitification. The median values of Li in fortified mushrooms corresponded to the fortification levels, increasing from 0.49 to 17 mg kg^−1^ dw relative to the background concentration of 0.056 mg kg^−1^ dw (control substrate contained 0.10 mg kg^−1^ dw). The potential for Li uptake in fruiting bodies was found to decrease at higher levels of fortification, with saturation occurring at 100 mg kg^−1^. Resulting lithiated mushrooms were up to 300-fold richer in Li content than specimens grown on control substrate. The fortification showed some effects on the uptake of other trace minerals, but concentrations of co-accumulated Ag, Al, As, Ba, Cd, Co, Cr, Cs, Cu, Hg, Mn, Ni, Pb, Rb, Sr, Tl, U, V and Zn were similar or lower than values reported in the literature for commercial *A. bisporus*. These lithiated mushrooms could be considered as a pro-medicinal alternative to treatments that use Li salts.

**Graphical abstract:**

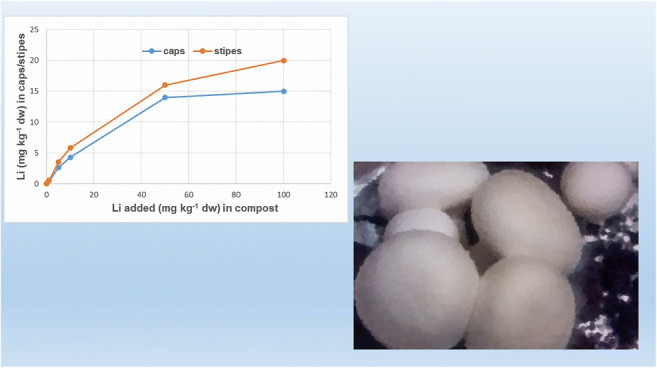

## Introduction

Wild edible mushrooms have been used since ancient times as nutritious and beneficial components of the diet of some individuals. An early example is the Shitake mushroom, *Lentinula edodes* (Berk.) Pegler, the first domesticated species in the ancient Ba and Shu states in the present province of Sichuan in China (Cetto, 2008). In general, mushrooms are good for bowel function due to a relatively high content of chitin-fibrous structures, but nutritionally, they also contain beneficial proteins, and bio-active organic and inorganic compounds including antioxidants such as glutathione, polysaccharides and pro-vitamins D and vitamin B complex (Jaworska et al. 2015; Muszyńska et al. 2017). They are relatively rich in potassium (K), phosphorous (P), nitrogen (N) and sulphur-containing compounds, and also in important micronutrients including Zn, Cu and Se (Falandysz and Borovička, 2013). On the other hand, under certain conditions, some edible wild and cultivated mushrooms were found to contain elevated concentrations of undesirable and detrimental toxic elements such as Ag, As, Cd, Hg, Pb, etc. or radioactive contamination in the form of alpha-emitting radionuclides and radiocaesium (^137^Cs), accumulated from their natural background or from anthropogenically polluted substrates (Borovička et al. 2010; Frank et al. 1974; Kavčič et al. 2019; Komorowicz et al. 2019; Strumińska-Parulska et al. 2020).

The discussion on whether lithium is biologically essential for human and animal growth and health functions continues (Anke et al. 2003; Aral and Vecchio-Sadus, 2008, Markert et al., 2018), but there have been few studies on its occurrence in fungi (Falandysz and Borovička, 2013). It is a minor mineral constituent of wild mushrooms, i.e. its concentration in edible fruit bodies and sclerotia is rarely more than 0.5 mg kg^−1^ dry weight (dw) (Drewnowska et al. 2017; Falandysz et al. 2017a; Mędyk et al. 2017; Mleczek et al. 2016; Vetter 2005). Recent studies (Zhang et al. 2020b) have shown that Li concentrations in wild mushrooms were 0.25 ± 0.06 mg kg^−1^ dw in *Boletus speciosus* Frost, 0.27 ± 0.09 mg kg^−1^ dw in *Boletus umbriniporus* Hongo and 0.27 ± 0.04 mg kg^−1^ dw in *Hemileccinum impolitum* (Fr.) Šutara (previous name *Boletus impolitus* Fr.)—all grown in soils with a polymetallic background in south-western China. A slightly smaller concentration of 0.022 ± 0.027 mg kg^−1^ dw (median 0.010 mg kg^−1^ dw) was observed in the sclerotia of the medicinal fungus *Wolfiporia cocos* (Schwein.) Ryvarden et Gilb., from SW China (Falandysz et al. 2017b). In Europe, Li concentrations of 0.09 ± 0.02 mg kg^−1^ dw in the Man on Horseback *Tricholoma equestre* (L.) P. Kumm. mushroom (Rzymski and Klimaszyk, 2018) and concentrations from 0.012 ± 0.003 to 0.075 ± 0.062 mg kg^−1^ dw in the caps of Fly Agaric *Amanita muscaria* (L.) Lam. have been reported (Falandysz et al. 2020).

Drugs containing Li in the form of carbonate, citrate, etc. are used to treat or prevent episodes of abnormal excitation or frenzied behaviour and mood disorders and, in high doses of 150 to 360 mg, to treat bipolar disorder (Marshall, 2015). However, the use of Li salts as drugs shows some side effects such as excessive urination, renal dysfunction, nausea, diarrhoea, thyroid and parathyroid gland dysfunction, neurological conditions (lethargy, ataxia, confusion, agitation), neuromuscular excitability (hyperreflexia, myoclonic jerks and muscle fasciculation), etc. (Rust et al. 2018). Additionally, Li_2_CO_3_ has been found to be poisonous above the maximal therapeutic dose (10.4 mg L^−1^ in blood) (Timmer and Sands, 1999). The debate on the relationship between increasing intakes of Li from municipal drinking water and a decrease in suicide rates (Marshall, 2015; Memon et al. 2020) continues, an approach that has been suggested but also questioned (Gillman, 2019; Ng et al. 2019; Szklarska and Rzymski, 2019). The active lithiation of foods and drinking water is highlighted as an area of current research aiming to increase the state of knowledge in this field (Szklarska and Rzymski, 2019). In the current climate of sustainability and natural sources of medicines, it is appropriate to consider alternative natural sources of Li, such as mushrooms, and the extent to which Li occurs in different species.

It is known from early experiments that mushroom growers may inadvertently influence the content of some mineral constituents accumulated in cultivated mushrooms, through a combination of substrate and cultivation method used, or simply by unintentional fortification of the growing substrate with soluble salts of toxic elements, e.g. Ag, As or Hg (Bressa et al. 1988; Falandysz et al. 1994; Huang and Xu 2014). Mycelia and fruit bodies (mushrooms) in the case of macromycetes can also be intentionally enriched by growers with minerals that are beneficial to human nutrition, e.g. Se or Zn, and some attempts have been made to test and develop fungal materials enriched in Li (Assunção et al. 2012; Faria et al. 2018; Klimaszewska et al. 2016; Muszyńska et al. 2015; Pankavec et al. 2021; Turło et al. 2007; Rodriquez Estrada et al. 2009; Rzymski et al. 2019). Assunção et al. (2012) demonstrated poor (or a lack of) accessibility of Li from Li_2_CO_3_ drugs, but much higher accessibility from lithiated *Pleurotus ostreatus* mushrooms in an in vitro digestibility experiment. The present study aimed to evaluate the accumulation of Li and co-accumulation of Ag, Al, As, Ba, Cd, Co, Cr, Cs, Cu, Hg, Mn, Ni, Pb, Rb, Sr, Tl, U, V and Zn in white *A. bisporus* mushrooms grown on commercial substrate that was fortified with the addition of Li in the form of the solubilized carbonate salt (Li_2_CO_3_).

## Materials and methods

### Substrate preparation and mushroom cultivation

The commercial set for the cultivation of *Agaricus bisporus* (J.E. Lange) Imbach used in this study contained a brand ready substrate inoculated with mycelium (8 kg of fermented substrate composed of straw and chicken manure; phase III) and a casing material (peat mixed with a portion of natural gypsum) in a special container (supplier information available at https://grzybyhobby.pl/). The substrate was uniformly watered with Li_2_CO_3_ dissolved in deionized water in order to homogenously disperse the salt solution. Each cultivation experiment with the added Li_2_CO_3_ at the assumed fortification level was performed in triplicate. In total, 21 substrate stands were prepared in parallel, providing three series of six and a control for each series. Each series of six stands was fortified with Li_2_CO_3_ solutions at concentrations of 1.0, 5.0, 10, 50, 100 and 500 mg kg^−1^ dw, and included one unfortified substrate as a control. After overgrowing with mycelium, the experimental substrates were further coated with a layer of casing material. This scheme used to cultivate *A. bisporus* followed a detailed set of instructions (temperature, humidity, sunlight, timing, etc.) from a local producer (available at https://grzybyhobby.pl/;https://grzybyhobby.pl/pieczarka-biaa/).

At the beginning of the cultivation experiment, concentrations of Li and other elements in the unfortified control substrates were determined by taking five subsamples of fresh substrate from each of the three control (reference) stands and pooled accordingly (3 pools each ca 100 g). The pooled samples were lyophilized, dry ground in a porcelain mortar and sieved through a 4-mm mesh plastic sieve to obtain a homogenous mixture. Each substrate sample was then transferred into a clean screw-capped polyethylene bottle (Wide-Mouth Opaque Amber HDPE Packaging Bottles with Caps, Thermo Scientific™ Nalgene™), and all three individual pools were sealed in a larger bag that was stored in dry and clean conditions until analysis. The Li concentration of this reference substrate was determined as 0.10 ± 0.00 mg kg^−1^ dw. The concentrations (mg kg^−1^ dw) of some of the elements determined in the reference substrate were as follows: Ag at < LOD, Al at 2.6 ± 0.0, As at 0.00024 ± 0.00001, Ba at 14 ± 0, Cd at < LOD, Co at 0.0000018 ± 0.0000001, Cr at 0.00019 ± 0.00001, Cs at 0.0021 ± 0.0000, Cu at 0.088 ± 0.001, Mn at 0.012 ± 0.000, Ni at 0.066 ± 0.001, Pb at 0.81 ± 0.01, Rb at 0.10 ± 0.00, Sr at 0.051 ± 0.003, Tl at 0.0026 ± 0.0000, U at 0.00039 ± 0.00001, V at 0.000082 ± 0.000004 and Zn at 0.0029 ± 0.0001.

In practice, commercial *A. bisporus* products that are sold fresh or pickled consist mostly of the caps (usually young with light pink to pink gills, and rarely contain dark brown to black hymenophores—these mature specimens are sometimes sold as a crude product) and upper part of the stem—a large portion of the lower stem is cut out and discarded. In this experiment, both the caps and almost the whole stems of the fruit bodies were used and examined, in order to (a) get an insight into the accumulation of Li and other elements, (b) understand the bio-concentration potential of Li and (c) evaluate the possible suitability of them as a lithiated product.

### Mushroom sample collection and preparation of the samples

**Fig. 1:**
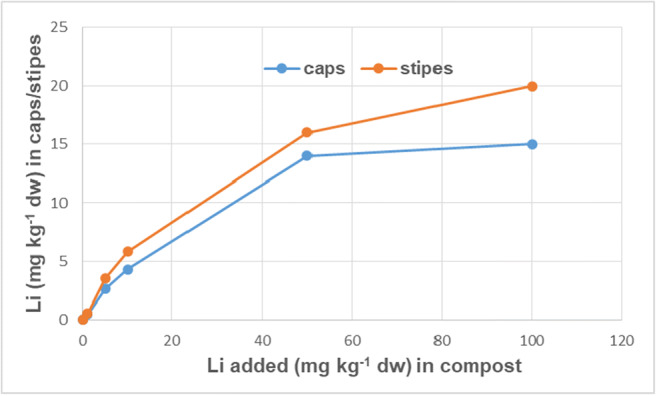
Graphical presentation of the dynamics of Li accumulation (median values) by *A. bisporus* from Li-fortified (Li_2_CO_3_) substrate

Mushrooms (Fig. 1) were collected at the first flush of growth, from all fortified and control stands. Around 60 of the emergent fruit bodies of appropriate size and maturity (young, at typical stage for high-quality commercial harvesting with light pink to pink gills) from each stand (yield around 1.1 kg per stand) were successively collected and cleaned from the peat debris using a ceramic knife and a brush. Fruit bodies from each pool were separated into caps and stems. The pooled subsample of the fungal materials obtained from each fortification experiment (24 pools of caps and 24 pools of stems, separately; *n* = 3 pooled samples with 50 fruit bodies per pool) at each level of Li_2_CO_3_ fortification were lyophilized, ground in a porcelain mortar and sieved through a 4-mm mesh plastic sieve to obtain a finely powdered product. Each sample of pooled powdered caps and stems from each fortification experiment was then transferred into a clean sealed polyethylene bag. All of the bags were placed in a larger bag that was sealed and stored in dry and clean condition until analysis.

### Analytics

The analytical methods used for separate determination of Hg and Li and other trace elements in mushrooms have been presented in detail before (Chudzińska et al. 2012; Drewnowska et al. 2017; Jarzyńska and Falandysz, 2011), but a summarized description is given below. Dehydrated and powdered fungal and substrate subsamples (~ 0.5 mg) were digested with 5 mL of 65% HNO_3_ (Suprapure, Merck, Germany) under pressure in a microwave oven (model Ethos One, Milestone Srl, Italy). The heating programme consisted of a single step, using a microwave power of 1500 W and a ramp time of 20 min to 200 °C, followed by a hold time of 30 min. Reagent blank solutions were prepared in the same way. For every set of 10 mushroom samples digested, two blank samples were run. The digest was further diluted to 10 mL using deionized water (TKA Smart2Pure, Niederelbert, Germany).

Elemental analysis of Ag, Al, As, Ba, Co, Cd, Cs, Cu, Cr, Li, Mn, Ni, Pb, Rb, Sr, V, Tl, U and Zn was carried out by inductively coupled plasma mass spectrometry (ICP-MS) using an ELAN DRC II (PerkinElmer, SCIEX, Canada), equipped with a Meinhard concentric nebulizer, cyclonic spray chamber, Pt cones and a quadruple mass analyser. Typical instrument operating conditions were RF power—1100 W; plasma argon (Ar) flow rate—15 L min^−1^; nebulizer Ar flow rate—0.87 L min^−1^; auxiliary Ar flow rate—1.2 L min^−1^ and lens voltage—7.5–9.0 V. A mixed standard solution with a concentration of 10 mg L^−1^ (Multielement Calibration Standard 3, Atomic Spectroscopy Standard, PerkinElmer Pure) was used for the construction of the calibration curve. In order to effectively correct temporal variations in signal intensity, the isotopes ^45^Sc, ^74^Ge, ^103^Rh and ^159^Tb (ICP Standard CertiPUR, Merck, Germany) prepared from individual solutions with a concentration of 1000 mg L^−1^ were applied as internal standards. Calibration curves for the elements were constructed in the range of 0.1 to 50 μg L^−1^. Argon with a purity of 99.999% was used as a nebulizer, auxiliary and plasma gas in ICP-MS (Messer, Chorzów, Poland).

Fungi can accumulate a range of mineral constituents in their fruiting bodies, and the resulting ash content of *A. bisporus* shows a median value of 9.35% (range from 9.23 to 9.48) (Muszyńska et al. 2017). Among the various minerals present in mushrooms, the element K is found at concentrations up to ~ 60,000 mg kg^−1^ dw, while so-called trace elements occur in a wide range with concentrations of the rare earths (Dy, Er, Eu, Tb, Tm, Lu) occurring at ~ 0.001 mg kg^−1^ dw (Falandysz and Borovička, 2013; Falandysz et al. 2001). Thus, the high mineral content and complexity of the mushroom matrix demand reliable and credible analytical methodology which includes robust quality control measures, particularly when a spectrum of elements has to be determined. This is essential for constructing baseline concentration datasets on occurrence and to assess the impact of anthropogenic factors, intake and risk/benefit for consumers.

The methods used were validated and controlled by preparation of standard solutions, calibration of instruments, running of blank samples and duplicates and replicates with each analytical cycle. In the case of Hg analysis, the samples were measured in triplicates and a blank was measured for every set of three samples of study material, and for the other elements, the blanks were measured with every set of ten samples. The certified reference materials and control materials analysed together with the fungal and substrate experimental material samples were CTA-OTL-1 (Polish Certified Reference Material For Multielement Trace Analysis: Oriental Tobacco Leaves) and CSM-2 (Control Material CS-M-2 As, Cd, Cr, Cu, Hg, Pb, Se and Zn in Dried Mushroom Powder - Champignon) produced by the Institute of Nuclear Chemistry and Technology at the Polish Academy of Sciences in Warsaw (Poland) and purchased from LGC STANDARDS Sp. z o.o. (Poland) (Table 1). Other control materials were also analysed, as described in previous reports (Falandysz et al. 2017a; Mędyk et al. 2017; Pankavec et al. 2021; Saba et al. 2016). The results were evaluated for quality prior to quantitation and reporting. Precision values for the elements were in the range 1–8%. The analytical recoveries based on the use of reference materials were typically in the following ranges (%): 90–119 for As, 98–113 for Cd, 86 for Co, 93 for Cu, 98 for Hg, 82 for Ni, 83–90 for Pb, 97 for Rb and 94 for Zn (Table 1). The detection and quantification limits (LOD and LOQ) for Hg were 0.003 and 0.005 mg kg^−1^dw, respectively. The LODs, calculated as three standard deviations of independent replicates of the reagent blank, were respectively (in μg L^−1^) for Ag, 0.003; As, 0.03; Ba, 0.2; Cd, 0.008; Co, 0.001; Cr, 0.2; Cu, 0.15; Li, 0.02; Mn, 0.4; Ni, 0.05; Pb, 0.07; Rb, 0.03; Sr, 0.06; Tl, 0.001; U, 0.004; V, 0.09; and Zn, 4.

**Table 1:** The certified and determined contents of trace elements (mg kg^−1^ dw) in certified reference material CTA-OTL-1 and control material CSM-2

Element	Certified value ± SD	Determined value ± SD	Recovery (%)
Material: CTA-OTL-1			
As	0.539 ± 0.060	0.47 ± 0.01	90.1
Cd	1.120 ± 0.12	1.3 ± 0.0	113.3
Co	0.879 ± 0.039	0.076 ± 0.034	86.5
Ni	6.32 ± 0.65	5.2 ± 0.3	82.2
Pb	4.91 ± 0.80	4.1 ± 0.0	82.6
Rb	9.79 ± 1.27	9.5 ± 0.4	97.3
Material: CSM-2			
As	0.151 ± 0.010	0.16 ± 0.07	119.3
Cd	0.092 ± 0.004	0.091 ± 0.011	98.5
Cr	1.205 ± 0.115	1.13 ± 0.05	93.4
Cu	22.90 ± 0.86	22.8 ± 2.1	99.7
Hg	0.164 ± 0.004	0.16 ± 0.01	98.5
Pb	0.111 ± 0.015	0.099 ± 0.015	89.9
Zn	42.50 ± 1.23	41 ± 1	94.5

The cap-to-stem concentration quotient values (index *Q*_C/S_) of accumulated Li were calculated using the median concentration values. A Pearson correlation was used to measure the strength of the linear association between the level of substrate fortification with Li and mass accumulation of Ag, Al, As, Ba, Co, Cd, Cs, Cu, Cr, Hg, Li, Mn, Ni, Pb, Rb, Sr, V, Tl, U and Zn in *A. bisporus*. A Mann-Whitney *U* test was used to detect any statistically significant difference in overall concentration of an element other than Li between the control and lithiated mushrooms, regardless of the lithiation level. A free, Social Science Statistics software (www.socscistatistics.com) and Microsoft Excel (2013 edition) were used for statistical analyses of the results and the drawing of graphs.

## Results and Discussion

### Lithium

The cultivation procedure that was used to grow *A. bisporus* mushrooms in commercial substrate proved to be successful in all of the test stands with effective production of fruiting bodies, except for the stand with the highest fortified Li concentration (500 mg kg^−1^). This level of fortification appeared to have totally inhibited fruiting. The controls showed that Li uptake from the unfortified substrate to the fruiting bodies reached a median concentration of 0.057 mg kg^−1^ dw in the caps and 0.055 mg kg^−1^ dw in the stems (estimated value of 0.056 mg kg^−1^ dw in the whole mushrooms, Table 2). The Li bio-concentration factors (BCF; expressed as the quotient of the concentration of an element in caps or stems to concentration level in the substrate, on dry to dry mass basis) associated with this uptake were 0.66 and 0.57, in the pools of the caps and stems, respectively. A BCF value of less than 1.0 is considered as evidence of bio-exclusion of an element by species. As the fortification levels of Li in the experimental substrate increased from 1.0 mg kg^−1^ dw, through to 5.0 mg kg^−1^ dw and 10 mg kg^−1^ dw, the potential of *A. bisporus* to bio-concentrate Li appears to have remained similar to that of the control substrate, i.e. BCF median values for mushrooms raised on the Li-fortified substrate were in the range from 0.43 to 0.51 for the caps and from 0.47 to 0.68 for the stems.

**Table 2:** Concentrations of Li (mg kg^−1^ dw) and BCFs in caps, stems and whole fruiting bodies of *A. bisporus* following uptake from fortified compost (mean ± standard deviation, median and range; *n* = 3 pooled samples for each fortification level)

Li^#^ mg kg^−1^ dry weight	Content of Li (mg kg^−1^ dry weight)			Content of Li (mg kg^−1^ wet weight)^*^			BCF_C_^*§^	BCF_s_
	Caps	Stems	Whole^**^	Caps	Stems	Whole^**^		
0	0.067 ± 0.019	0.057 ± 0.008	0.062 ± 0.014	0.0063 ± 0.0018	0.0054 ± 0.0007	0.0059 ± 0.0013	0.67 ± 0.01	0.56 ± 0.01
	0.057	0.055	0.056	0.0054	0.0052	0.0051	0.66	0.57
	0.054–0.089	0.050–0.066	0.052–0.079	0.0051–0.0084	0.0047–0.0062	0.0049–0.0074	0.64–0.68	0.55–0.58
1.0	0.32 ± 0.28	0.40 ± 0.35	0.36 ± 0.31	0.030 ± 0.026	0.038 ± 0.033	0.034 ± 0.029	0.29 ± 0.24	0.36 ± 0.24
	0.47	0.52	0.49	0.044	0.049	0.046	0.43	0.47
	0.47–0.50	0.52–0.68	0.49–0.58	0.044–0.047	0.049–0.064	0.046–0.054	0.43–0.45	0.47–0.62
5.0	2.7 ± 0.5	3.5 ± 0.8	3.1 ± 0.6	0.25 ± 0.05	0.33 ± 0.07	0.29 ± 0.06	0.52 ± 0.11	0.68 ± 0.02
	2.6	3.5	3.0	0.24	0.33	0.28	0.51	0.68
	2.3–3.2	3.0–4.1	2.6–3.6	0.22–0.30	0.28–0.38	0.24–0.34	0.45–0.63	0.59–0.80
10	5.2 ± 1.5	6.7 ± 1.6	5.9 ± 1.5	0.49 ± 0.14	630 ± 150	550 ± 140	0.51 ± 0.14	0.66 ± 0.15
	4.3	5.8	5.0	0.40	0.54	0.47	0.43	0.58
	4.3–6.9	5.7–8.5	4.9–7.6	0.40–0.65	0.54–0.80	0.46–0.71	0.42–0.68	0.56–0.84
50	17 ± 11	24 ± 16	20 ± 13	1.6 ± 1.0	2.3 ± 1.5	1.9 ± 1.2	0.34 ± 0.21	0.47 ± 0.31
	14	16	15	1.3	1.5	1.4	0.14	0.16
	8.8–29	14–42	11–35	0.83–2.7	1.3–3.9	1.0–3.3	0.17- 0.57	0.03–0.83
100	14 ± 5	17 ± 5	15 ± 5	1.3 ± 0.5	1.6 ± 0.5	1.4 ± 0.5	0.14 ± 0.05	0.17 ± 0.05
	15	20	17	1.4	1.9	1.6	0.15	0.20
	8.0–18	11–21	9.3–19	0.75–1.7	1.0–2.0	0.88–1.8	0.08–1.8	0.11–0.21

^#^Fortification level in compost

^*^Calculated from dry weight data–dry matter content at 9.49%, range 9.37 ± 1.43–9.62 ± 1.04% (Vetter, 2003)

^**^The mean share of the biomass of the caps and stems (percentage by mass) in the whole fruiting bodies was 55:45

^§^Baseline Li content in compost was 0.10 ± 0.00 mg kg^−1^ dw

As the level of substrate fortification increased (1.0, 5.0 and 10 mg kg^−1^ dw), the resulting concentrations of accumulated Li also increased in the mushrooms, reaching median concentrations of 0.47, 2.6 and 4.3 mg kg^−1^ dw in the caps and 0.52, 3.5 and 5.8 mg kg^−1^ dw in the stems, respectively (Table 2). Further increases in the levels of fortification to 50 and 100 mg kg^−1^ dw also showed corresponding increases in lithiation (median values were 14 and 15 mg kg^−1^ dw in the caps, and 16 and 20 mg kg^−1^ dw in the stems, respectively). However, at these higher levels of substrate fortification (50 and 100 mg kg^−1^ dw), the potential of *A. bisporus* to bio-concentrate Li appears to have substantially decreased, showing median values of the BCF at 0.14 and 0.15 for the caps and 0.16 and 0.20 for the stems (Table 2).

The *Q*_C/S_ was 0.96 for the control *A. bisporus* and 1.1, 1.3, 1.3, 1.1 and 1.3, respectively, for the fruiting bodies, corresponding to the fortification levels of 1.0, 5.0, 10, 50 and 100 mg kg^−1^ dw. Thus, the redistribution of Li absorbed by mycelium of *A. bisporus*, between the stem and cap, remained unchanged, regardless of the fortification level of the substrate or the accumulated amount of the element, which ranged from 0.47 to 15 mg kg^−1^ dw in the caps and 0.52 to 20 mg kg^−1^ dw in the stems.

The results indicate that the addition of Li in the form of Li_2_CO_3_ to the substrate at concentrations up to 100 mg kg^−1^ dw resulted in a dose-dependent increase in the accumulation of this element [*r* = 0.93 for caps (result was significant at *p* < 0.01; the coefficient of determination was 86%) and *r* = 0.97 for stems (result was significant at *p* < 0.01; the coefficient of determination was 91%)]. A plot of these data (Fig. 1) also shows that these dependencies appear to level off as the fortification level approaches 100 mg kg^−1^, suggesting that “saturation” of the fruit bodies occurs at this concentration. The higher fortification level of 500 mg kg^−1^ dw appears to be detrimental to growth, as this fortified stand showed no evidence of fruiting. However, fortification levels of up to 100 mg kg^−1^ dw did not show any effect on biomass growth (no stimulation) in relation to the control. These results therefore show that fortification of the substrate with Li_2_CO_3_ may be used as a method to produce lithiated *A. bisporus* mushrooms at concentrations of up to 300 times the background concentrations.

Mleczek et al. (2016) attempted to bio-fortify *Ganoderma lucidum*, *Pleurotus eryngii* and *Pleurotus ostreatus* mushrooms by using Li_2_CO_3_- and CH_3_COOLi-fortified experimental substrates. The study reported that Li was better available from Li_2_CO_3_ then CH_3_COOLi, and that *G. lucidum* was a more efficient accumulator of Li than *P. eryngii* and *P. ostreatus* (Mleczek et al. (2016).

### Copper, manganese and zinc

The elements Cu, Mn and Zn are micronutrients in fungi and are important mineral constituents of the human diet. They are taken up by mycelia to meet the physiological requirements of the species, and their availability for fungi is typically maintained through natural occurrence of these elements in soil or other fungal substrates. A high excess of these mineral micronutrients in soil due to anthropogenic pollution, both regional and local, e.g. with Cu and Zn from copper/zinc ore and scrap metal smelters and foundries, can cause elevated concentrations of Cu and Zn in mushrooms. Other toxic metals such as Cd, Hg, Pb, etc. that are also typically co-emitted from some of the same sources can also bio-accumulate in mushrooms (Barcan et al. 1998; Falandysz, 2017; Zimmermannova et al., 2001).

The median Cu concentrations in the control mushrooms in this study were 18 mg kg^−1^ dw in the caps and 12 mg kg^−1^ dw in the stems, and mushrooms grown in the Li-fortified substrate showed similar median values in the caps (range 15 to 26 mg kg^−1^ dw, *p* > 0.05, M-W *U* test), with higher overall concentrations in the stems (13 to 19 mg kg^−1^ dw, *p* < 0.05, M-W *U* test), respectively (Table 3). The mushrooms that grew in the substrate fortified with Li at the levels of 1.0 and 5.0 kg^−1^ dw accumulated slightly more Cu than those in the control group (*p* < 0.05). Nonetheless, this increase in the concentration of Cu in the stems did not correlate with the level of substrate fortification with Li (*p* > 0.05, Pearson).

**Table 3:** Concentration of Ag, Al, As, Ba, Cd, Co, Cr, Cs, Cu, Hg, Mn, Ni, Pb, Rb, Sr, Tl, U, V and Zn (mg kg^−1^ dw; mean, standard deviation, median and range) in caps and stems of fruiting bodies of *A. bisporus* grown in substrate fortified with Li_2_CO_3_ at levels of 0, 1.0, 5.0, 10, 50 and 100 mg kg^−1^ in the dried compost. Mean, the standard deviation (± SD), median and the values range (minimum–maximum) follow the results

Parameter	Fortification level with Li, morphological part and corresponding content of elements (mg kg^−1^ dry matter)											
	0 (control)		1.0		5.0		10		50		100	
	Caps	Stems	Caps	Stems	Caps	Stems	Caps	Stems	Caps	Stems	Caps	Stems
Ag	0.08 ± 0.02	0.03 ± 0.01	0.09 ± 0.01	0.07 ± 0.01	0.09 ± 0.01	0.31 ± 0.42	0.07 ± 0.03	0.06 ± 0.01	0.06 ± 0.01	0.03 ± 0.02	0.07 ± 0.02	0.05 ± 0.02
	0.07	0.03	0.09	0.07	0.09	0.07	0.05	0.06	0.06	0.03	0.06	0.04
	0.07–0.11	0.02–0.05	0.09–0.11	0.07–0.08	0.07–0.11	0.04–0.79	0.04–0.11	0.05–0.07	0.05–0.06	0.01–0.05	0.06–0.09	0.03–0.07
Al	0.96 ± 0.28	1.4 ± 0.1	1.4 ± 0.5	3.2 ± 1.3	1.5 ± 0.4	5.3 ± 4.9	1.5 ± 1.1	2.4 ± 0.7	1.6 ± 0.7	2.3 ± 0.6	1.8 ± 1.3	1.7 ± 0.4
	0.87	1.4	1.4	3.2	1.5	2.6	0.90	2.2	1.6	2.6	1.1	1.5
	0.74–1.3	1.2–1.5	1.0–1.8	2.3–4.1	1.1–1.9	2.5–11	0.85–2.8	1.8–3.2	0.91–2.3	1.5–2.6	0.92–3.2	1.5–2.3
As	0.17 ± 0.01	0.12 ± 0.02	0.25 ± 0.02	0.15 ± 0.04	0.24 ± 0.04	0.48 ± 0.58	0.23 ± 0.03	0.11 ± 0.01	0.16 ± 0.01	0.15 ± 0.11	0.24 ± 0.04	0.14 ± 0.02
	0.17	0.11	0.26	0.15	0.24	0.16	0.24	0.10	0.15	0.10	0.25	0.15
	0.16–0.18	0.09–0.13	0.24–0.27	0.12–0.18	0.19–0.29	0.12–1.1	0.20–0.25	0.09–0.11	0.15–0.18	0.08–0.27	0.19–0.26	0.11–0.15
Ba	0.56 ± 0.56	< LOD	< LOD	< LOD	0.75 ± 0.20	< LOD	0.56 ± 0.14	0.98 ± 0.50	0.44 ± 0.31	0.49 ± 0.27	0.79 ± 0.47	< LOD
	0.56				0.72		0.56	0.79	0.28	0.38	0.92	
	0.16–0.96				0.57–0.97		0.46–0.67	0.61–1.5	0.24–0.81	0.31–0.81	0.27–1.2	
Cd	0.14 ± 0.03	0.08 ± 0.01	0.22 ± 0.06	0.14 ± 0.02	0.18 ± 0.04	0.34 ± 0.37	0.18 ± 0.06	0.10 ± 0.04	0.11 ± 0.02	0.12 ± 0.07	0.19 ± 0.05	0.12 ± 0.03
	0.12	0.08	0.22	0.14	0.18	0.15	0.15	0.09	0.10	0.09	0.18	0.12
	0.12–0.18	0.07–0.09	0.17–0.26	0.12–0.15	0.13–0.21	0.10–0.78	0.13–0.25	0.06–0.15	0.10–0.13	0.06–0.20	0.16–0.24	0.09–0.15
Co	0.01 ± 0.01	0.003 ± 0.0009	0.0009 ± 0.0005	0.002 ± 0.001	0.003 ± 0.002	0.005 ± 0.005	0.007 ± 0.006	0.005 ± 0.004	0.002 ± 0.001	0.002 ± 0.001	0.002 ± 0.0003	0.001 ± 0.000
	0.001	0.002	0.0009	0.002	0.004	0.003	0.005	0.003	0.003	0.002	0.002	0.0013
	0.0009–0.03	0.002–0.004	0.0005–0.001	0.001–0.002	0.002–0.004	0.001–0.012	0.001–0.015	0.002–0.010	0.001–0.004	0.001–0.003	0.0018–0.0025	0.0010–0.0016
Cr	0.009 ± 0.001	0.009 ± 0.003	0.011 ± 0.006	0.011 ± 0.008	0.010 ± 0.004	0.025 ± 0.018	0.01 ± 0.01	0.01 ± 0.00	0.01 ± 0.01	0.008 ± 0.002	0.01 ± 0.00	0.008 ± 0.005
	0.009	0.007	0.011	0.011	0.010	0.016	0.012	0.009	0.008	0.008	0.01	0.007
	0.008–0.009	0.007–0.013	0.007–0.016	0.005–0.017	0.006–0.014	0.01–0.05	0.006–0.012	0.007–0.015	0.008–0.017	0.006–0.011	0.010–0.011	0.004–0.014
Cs	0.025 ± 0.002	0.018 ± 0.002	0.034 ± 0.002	0.023 ± 0.001	0.033 ± 0.001	0.07 ± 0.08	0.033 ± 0.005	0.018 ± 0.003	0.022 ± 0.001	0.021 ± 0.009	0.032 ± 0.004	0.022 ± 0.004
	0.025	0.017	0.034	0.023	0.032	0.02	0.032	0.018	0.023	0.017	0.032	0.021
	0.023–0.028	0.015–0.021	0.033–0.036	0.022–0.024	0.032–0.034	0.02–0.17	0.028–0.039	0.015–0.021	0.021–0.024	0.015–0.033	0.029–0.037	0.018–0.026
Cu	18 ± 1	12 ± 1	26 ± 7	17 ± 4	23 ± 4	55 ± 66	22 ± 3	15 ± 2	15 ± 1	14 ± 4	21 ± 1	15 ± 1
	18	12	26	17	21	19	23	16	15	13	21	16
	17–20	11–13	21–31	14–20	20–28	14–130	18–24	12–17	13–16	11–18	21–22	14–16
Hg	0.078 ± 0.003	0.059 ± 0.003	0.073 ± 0.011	0.053 ± 0.005	0.066 ± 0.006	0.043 ± 0.006	0.066 ± 0.011	0.046 ± 0.009	0.070 ± 0.019	0.053 ± 0.017	0.062 ± 0.012	0.045 ± 0.009
	0.078	0.059	0.073	0.054	0.063	0.043	0.059	0.042	0.066	0.046	0.056	0.041
	0.075–0.082	0.056–0.062	0.065–0.081	0.049–0.057	0.052–0.065	0.037–0.048	0.059–0.079	0.039–0.056	0.053–0.092	0.041–0.073	0.055–0.076	0.039–0.057
Mn	5.2 ± 0.7	4.3 ± 0.8	4.8 ± 0.1	3.9 ± 0.6	4.5 ± 0.4	12 ± 14	4.7 ± 0.5	3.3 ± 0.3	3.8 ± 0.8	3.8 ± 0.5	4.9 ± 0.1	3.6 ± 0.2
	5.5	4.2	4.8	3.9	4.67	3.9	4.6	3.3	3.6	3.7	4.8	3.6
	4.4–5.6	3.5–5.1	7.8–4.9	3.5–4.3	4.1–4.8	3.7–29	4.2–5.2	3.1–3.7	3.2–4.8	3.2–4.3	4.8–5.0	3.4–3.9
Ni	0.026 ± 0.010	0.023 ± 0.007	0.017 ± 0.011	0.018 ± 0.017	0.037 ± 0.011	0.095 ± 0.11	0.015 ± 0.009	0.030 ± 0.012	0.48 ± 0.81	0.020 ± 0.008	0.025 ± 0.013	0.052 ± 0.029
	0.027	0.022	0.017	0.018	0.031	0.054	0.012	0.037	0.018	0.022	0.021	0.056
	0.014–0.038	0.017–0.031	0.009–0.025	0.006–0.031	0.030–0.051	0.009–0.22	0.007–0.025	0.016–0.037	0.011–1.4	0.010–0.027	0.014–0.039	0.021–0.079
Pb	0.10 ± 0.15	0.046 ± 0.039	0.015 ± 0.006	0.017 ± 0.004	0.041 ± 0.017	0.048 ± 0.050	0.15 ± 0.12	0.048 ± 0.047	0.056 ± 0.034	0.024 ± 0.011	0.021 ± 0.005	0.038 ± 0.029
	0.022	0.035	0.015	0.017	0.039	0.029	0.19	0.026	0.053	0.022	0.023	0.028
	0.011–0.27	0.014–0.089	0.011–0.019	0.014–0.019	0.025–0.061	0.010–0.11	0.013–0.24	0.014–0.102	0.024–0.093	0.016–0.036	0.016–0.025	0.015–0.070
Rb	8.6 ± 0.7	5.9 ± 0.6	12 ± 1	7.4 ± 1.6	11 ± 1	24 ± 29	11 ± 1	6.3 ± 1.0	7.7 ± 0.5	7.7 ± 3.9	12 ± 1	7.0 ± 0.4
	8.7	5.6	12	7.4	11	8.2	11	6.7	7.5	6.1	13	6.8
	7.8–9.3	5.5–6.6	11–12	6.3–8.6	10–13	6.1–57	9.9–13	5.2–6.9	7.4–8.3	4.9–12	11–13	6.7–7.4
Sr	0.22 ± 0.07	0.68 ± 0.21	0.28 ± 0.09	0.88 ± 0.02	0.22 ± 0.50	3.3 ± 4.5	0.29 ± 0.17	0.91 ± 0.05	0.47 ± 0.29	0.66 ± 0.42	0.32 ± 0.15	1.0–0.5
	0.26	0.59	0.28	0.88	0.24	0.71	0.23	0.93	0.42	0.76	0.37	1.3
	0.14–0.27	0.53–0.92	0.22–0.35	0.87–0.90	0.17–0.26	0.68–8.5	0.15–0.48	0.85–0.95	0.19–0.78	0.20–1.0	0.14–0.45	0.43–1.4
Tl	0.006 ± 0.001	0.006 ± 0.001	0.005 ± 0.001	0.004 ± 0.001	0.006 ± 0.002	0.025 ± 0.04	0.005 ± 0.002	0.004 ± 0.002	0.005 ± 0.001	0.005 ± 0.001	0.005 ± 0.001	0.004 ± 0.001
	0.006	0.005	0.005	0.004	0.006	0.006	0.004	0.004	0.006	0.005	0.005	0.004
	0.005–0.006	0.005–0.006	0.004–0.005	0.003–0.005	0.004–0.008	0.003–0.067	0.004–0.007	0.003–0.007	0.004–0.006	0.004–0.005	0.003–0.006	0.003–0.005
U	0.0002 ± 0.0001	0.001 ± 0.0004	0.0003 ± 0.0003	0.0010 ± 0.0001	0.0004 ± 0.0002	0.0025 ± 0.0035	0.0004 ± 0.0006	0.0010 ± 0.0005	0.0005 ± 0.0006	0.0008 ± 0.0006	0.0005 ± 0.0004	0.0008 ± 0.0005
	0.0002	0.0009	0.0003	0.001	0.0005	0.0008	0.0001	0.0007	0.0003	0.0009	0.0005	0.0011
	0.0001–0.0003	0.0008–0.0015	0.0001–0.0005	0.0009–0.0011	0.0002–0.0006	0.0002–0.0066	0.0001–0.0011	0.0006–0.0016	0.0001–0.0013	0.0001–0.0013	0.0002–0.0008	0.0003–0.0012
V	0.0029 ± 0.0006	0.0058 ± 0.0007	0.0035 ± 0.002	0.0056 ± 0.0024	0.0037 ± 0.0012	0.017 ± 0.016	0.0046 ± 0.0036	0.0071 ± 0.0009	0.0028 ± 0.0019	0.0057 ± 0.0026	0.0022 ± 0.0006	0.0066 ± 0.0018
	0.0027	0.0062	0.0035	0.0056	0.0039	0.009	0.0026	0.0070	0.0033	0.0069	0.0019	0.0071
	0.0025–0.0036	0.0049–0.0063	0.0022–0.0048	0.0039–0.0072	0.0024–0.0064	0.0065–0.036	0.0026–0.0088	0.0063–0.0081	0.0007–0.0044	0.0027–0.0075	0.0017–0.0028	0.0046–0.0083
Zn	43 ± 3	35 ± 0	47 ± 1	37 ± 7	44 ± 6	120 ± 150	45 ± 4	30 ± 3	40 ± 6	38 ± 8	46 ± 1	35 ± 2
	42	35	47	37	41	40	44	31	38	38	46	36
	41–46	35–35	47–48	32–42	39–51	31–290	41–49	26–33	37–46	31–46	45–47	33–37

< LOD (limit of detection)

The Cu concentrations determined in the *A. bisporus* mushrooms in this study were closer to the lower range of results (mean 36 ± 14 mg kg^−1^ dw, and total range from 13 to 75 mg kg^−1^ dw; *n* = 72 fruit bodies) and 65 ± 9 mg kg^−1^ dw (*n* = 15) published for the white strain of button mushroom available from retail outlets in Poland, during 1989 and 2007–2015, respectively (Falandysz et al. 1993; Pankavec et al. 2019; Rzymski et al. 2017), and similarly, for the Australian cultivated white button mushrooms from two producers (range of the means from 35 ± 2 to 61 ± 2 mg kg^−1^ dw in 6 pools of an unknown number of fruiting bodies) (Koyyalamudi et al. 2013). The authors Falandysz et al. (1993), Muszyńska et al. (2017), Rzymski et al. (2017) and Vetter et al. (2005) reviewed data on Cu in *A. bisporus* and presented values in the range from 25 to 125 mg kg^−1^ dw, from 3 to 65 mg kg^−1^ dw and from 6.8 to 61 mg kg^−1^ dw, respectively (no information was provided on the analytical quality of compiled data and sample size information was not included).

The median Mn concentrations in the control *A. bisporus* were 5.5 mg kg^−1^ dw in the caps and 4.2 mg kg^−1^ dw in the stems, and mushrooms grown in the Li-fortified substrate showed significant statistically lower median values (range from 3.6 to 4.8 mg kg^−1^ dw (*p* < 0.05, M-W *U* test), and from 3.3 to 3.9 mg kg^−1^ dw (*p* < 0.05; M-W *U* test), respectively (Table 3). The decrease of Mn in lithiated mushrooms did not correlate to the level of substrate fortification for either caps or stems (*p* > 0.05; Pearson).

The observed concentrations in this study seem typical for this species (cultivated), when compared to the majority of values reviewed by Vetter et al. (2005). Manganese occurred in the range from 7.7 ± 0.2 to 21 ± 1 mg kg^−1^ dw in white *A. bisporus* studied by Koyyalamudi et al. (2013), and was reported at a mean concentration of 5.9 ± 0.6 mg kg^−1^ dw (Falandysz et al. 1993) and 6.1 ± 2.0 mg kg^−1^ dw (total range 4.1 to 12 mg kg^−1^ dw) in the study by Rzymski et al. (2017). The review by Vetter et al. (2005) had quoted Mn in cultivated white button mushrooms in the range from 3 to 11 mg kg^−1^ dw, with an outlying concentration of 35 mg kg^−1^ dw. Thus, the concentrations of Mn in *A. bisporus* grown in the control and Li-fortified substrate in this study corresponded to the lower range of Mn concentrations reported for this species in the literature.

The median Zn concentrations in the control *A. bisporus* were 42 mg kg^−1^ dw in the caps and 35 mg kg^−1^ dw in the stems. These were similar to the median Zn values for mushrooms grown in the Li-fortified substrate which ranged from 38 to 42 mg kg^−1^ dw (*p* > 0.05, both M-W *U* test and Pearson) in the caps and from 31 to 40 mg kg^−1^ dw (*p* > 0.05, both M-W *U* test and Pearson) in the stems (Table 3).

These concentrations can be considered as typical for cultivated *A. bisporus*. Koyyalamudi et al. (2013) studied Zn in *A. bisporus* fruiting bodies in three flushes, collected from two different farms. They observed a substantial variation in concentrations between the flushes (mushrooms from the first flush showed higher concentrations) with an overall range from 27 ± 0 to 44 ± 0 mg kg^−1^ dw (figures rounded). Whole *A. bisporus* from a Polish market showed a Zn concentration of 100 ± 15 mg kg^−1^ dw (Falandysz et al. 1993) and 66 ± 14 mg kg^−1^ dw; the totals ranged from 41 to 95 mg kg^−1^ dw (Rzymski et al. 2017). As expected, the range of values reported for Zn in cultivated *A. bisporus* is relatively wide, ranging from 4.8 to 110 mg kg^−1^ dw, as reported in a compilation by Muszyńska et al. (2017) and from 14 to 210 mg kg^−1^ dw in the study by Vetter et al. (2005).

### The elements Al, Ba, Co, Cs, Ni, Rb, Sr and V

The median values of Al concentrations in the lithiated *A. bisporus* were greater (*p* < 0.05, M-W *U* test) overall, both in the caps (0.90 to 1.6 mg kg^−1^ dw) and in the stems (1.5 to 3.2 mg kg^−1^ dw) when compared to the control caps (0.87 mg kg^−1^ dw) and stems (1.4 mg kg^−1^ dw), and were independent of the level of Li fortification (*p* > 0.05, Pearson) (Table 3). These results are substantially lower than the value of 14 ± 0 mg kg^−1^ dw reported for retail samples of *A. bisporus* (*n* = 15) by Müller et al. (1997).

Al is a common but trace constituent of mushrooms. Wild edible mushrooms appear to accumulate more Al than *A. bisporus*, and occurrence has been reported in several popular species, including the King Bolete *Boletus edulis* (34 ± 24 mg kg^−1^ dw) or the Brown Birch Scaber Stalk *Leccinum scabrum* (median value range from 30 to 40 mg kg^−1^ dw) (Mędyk et al. 2020; Müller et al. 1997). The authors Stijve et al. (2004) have proved that contamination of mushrooms with adhering soil and sand particles, which is often very hard or impossible to avoid, is a major source of error in the results of determination of Al and several other chemical elements. Typically, this is caused by soil/particulate contamination on the surfaces of fruit bodies. In order to avoid unreliable results for major soil elements (Al, Fe, Ca), but also for vanadium and the lanthanides, a perfect cleanup from adhered debris using a plastic or ceramic knife and/or plastic brush (a portion of a stem can be carefully scraped and bottom part cut off—which is a common practice by recreational or commercial foragers) is a crucial step before any further sample treatment. Rinsing with deionized water can also be effective, but intensive washing and soaking can deplete some metal components. Washing, and washing in parallel with intensive brushing of relatively hard mushrooms, is common culinary practice*.*

The median Ba concentration in the control *A. bisporus* mushrooms was 0.56 mg kg^−1^ dw in the caps (data < LOD for stems), and mushrooms grown in the Li-fortified substrate showed median values ranging from 0.28 to 0.92 mg kg^−1^ dw in the caps (*p* > 0.05; Pearson), and from 0.38 to 0.79 mg kg^−1^ dw in the stems (Table 3). Barium is not considered as a micronutrient for fungi and is passively co-accumulated with other alkali earth elements such as Ca and Mg which are more abundant in fruiting bodies, with concentrations of around 500 to 2000 mg kg^−1^ dw and around 1000 to 1500 mg kg^−1^ dw, respectively, reported for *A. bisporus* (Falandysz and Borovička, 2013; Koyyalamudi et al. 2013; Rzymski et al. 2017) (Table 3).

The median Co concentrations in the control *A. bisporus* mushrooms were 0.001 mg kg^−1^ dw in the caps and 0.002 mg kg^−1^ dw in the stems, and mushrooms grown in the Li-fortified substrate showed median concentrations ranging from 0.0009 to 0.005 mg kg^−1^ dw (*p* > 0.05, both M-W *U* test and Pearson), and from 0.0013 to 0.003 mg kg^−1^ dw (*p* > 0.05, both M-W *U* test and Pearson), in caps and stems, respectively (Table 3). Reported concentrations of Co in *A. bisporus* ranged from 0.095 to 0.37 mg kg^−1^ dw (Koyyalamudi et al. 2013). This element is a very minor micronutrient in edible mushrooms, and wild species seem to be richer than cultivated *A. bisporus*, e.g. a Co concentration of 0.13 ± 0.09 mg kg^−1^ dw was reported in the edible caps of *Macrolepiota procera* (Parasol Mushroom) in Europe (Falandysz et al. 2017d). Concentrations from 0.28 to 2.5 mg kg^−1^ dw were reported in the caps of the *Hemileccinum impolitum*, *Butyriboletus roseoflavus* and *Boletus umbriniporus* from Yunnan in China (Zhang et al. 2020b), and 0.68 to 1.2 mg kg^−1^ dw were reported in the caps of *Suillellus luridus* (previous name *Boletus luridus*), *Boletus magnificus* and *Boletus tomentipes* also from Yunnan (Falandysz et al. 2017c).

The median Cs concentrations in the control *A. bisporus* were 0.025 mg kg^−1^ dw in the caps and 0.017 mg kg^−1^ in the stems, and mushrooms grown in the Li-fortified substrate showed median values in the range from 0.023 to 0.034 mg kg^−1^ dw (*p* < 0.05, M-W *U* test; *p* > 0.05, Pearson), and from 0.017 to 0.023 mg kg^−1^ dw (*p* < 0.05, M-W *U* test; *p* > 0.05, Pearson), in caps and stems, respectively (Table 3). No original data could be identified regarding the occurrence of Cs in cultivated *A. bisporus* in the available literature. Caesium is a minor element in *A. bisporus* when compared to the alkali elements such as Li or Rb in this study (Tables 2 and 3), and concentrations are insignificant when compared to sodium (Na) and potassium (K), which were reported in the range from 760 to 860 mg kg^−1^ dw and from 35,000 to 45,200 mg kg^−1^ dw, respectively, as reviewed by Muszyńska et al. (2017). In the present study, lithiation of *A. bisporus* did not show any effect on the co-accumulation of Cs.

Median Ni concentrations in the control *A. bisporus* were 0.027 mg kg^−1^ dw in the caps and 0.022 mg kg^−1^ dw in the stems, and mushrooms grown in the Li-fortified substrate showed median values in the range from 0.012 to 0.031 mg kg^−1^ dw (*p* > 0.05, both M-W *U* test and Pearson), and from 0.018 to 0.056 mg kg^−1^ dw (*p* > 0.05, both M-W *U* test and Pearson), in caps and stems, respectively (Table 3). Nickel is not considered as a micronutrient in fungi. The element is generally emitted from anthropogenic sources, e.g. from Ni-Cu smelters. Mushrooms foraged in an area of 3000 km^2^ around a smelter were reported to accumulate Ni in a dose (concentration in soil)-dependent (concentration in fruiting bodies) manner, with concentrations at 15- to 40-fold above background levels (Barcan et al. 1998).

The yellow-cracking Bolete *Xerocomus subtomentosus*, sampled over three successive years from the same background area, showed median Ni concentrations in the range from 0.46 to 0.7 mg kg^−1^ dw in the caps (total range 0.35 to 1.0 mg kg^−1^ dw) (Chojnacka et al. 2013), while in another study, the same species collected at a distance of 11 to 165 km from a Ni-Cu smelter complex showed Ni concentrations in the whole fruit bodies in the range from 5 to 19 mg kg^−1^ dw (Barcan et al. 1998). In a recent study (Rzymski et al. 2017), Ni occurrence has been reported in batches of white *A. bisporus* from retail outlets in Poland, with concentrations that were up to two orders of magnitude greater than those found in the present study (Table 3), i.e. the mean concentration was 1.1 ± 1.4 mg kg^−1^ dw (range from 0.08 to as high as 6.2 mg kg^−1^ dw).

Median Rb concentrations in the control *A. bisporus* mushroom were 8.7 mg kg^−1^ dw in the caps and 5.6 mg kg^−1^ dw in the stems, and mushrooms grown in the Li-fortified substrate showed median values in the range from 7.5 to 13 mg kg^−1^ dw in the caps (*p* > 0.05, both M-W *U* test and Pearson) and from 6.1 to 8.2 mg kg^−1^ dw in the stems (*p* < 0.05, M-W *U* test and *p* > 0.05, Pearson) (Table 3).

After potassium (K), the element Rb is the second most abundant alkali element in mushrooms. It is distributed at around a twofold greater concentration in the caps than in the stems of the fruiting bodies, e.g. the reported mean was 780 ± 230 (median 750 and range 330 to 1200) mg kg^−1^ dw in the caps of *S. imbricatus* (Saba et al. 2020), and ranged from 330 ± 200 to 410 ± 110 mg kg^−1^ dw (total range 120 to 760 mg kg^−1^ dw) in the caps of *L. scabrum* (Mędyk et al. 2020). Despite its relative abundance in fungal fruiting bodies, Rb is not considered as an essential element in fungi. Its accumulation, e.g. in the caps and stems of the fruiting bodies of Fly Agaric (*A. muscaria*), is different from that of potassium, for which Rb is a close chemical analogue (Falandysz et al. 2020).

The median Sr concentration in the control *A. bisporus* caps was 0.26 mg kg^−1^ dw, and ranged from 0.23 to 0.42 mg kg^−1^ dw in the lithiated specimens (*p* > 0.05, both M-W *U* test and Pearson) (Table 3). The Sr concentration in the stems of the non-lithiated mushrooms at a median value of 0.59 mg kg^−1^ dw was statistically lower than that of the lithiated specimens (range from 0.71 to 1.3 mg kg^−1^ dw, *p* < 0.05, M-W *U* test), and independent of the level of fortification (*p* > 0.05, Pearson) (Table 3).

Strontium is weakly accumulated in the fruiting bodies of popular wild fungi, and its BCF values are usually well below one. For example, in popular edible species such as *B. edulis*, the BCFs were 0.06 ± 0.02 (0.05 to 0.09) for caps and higher for stems at 0.14 ± 0.08 (0.07 to 0.25), while the median concentrations in *X. subtomentosus* ranged from 0.06 to 0.83 in the caps and from 0.13 to 1.3 in the stems (Chojnacka et al. 2013; Frankowska et al. 2010). Data on Sr in mushrooms and in parallel, in the underlying soil, are rare in the literature. Řanda and Kučera (2004) have reported a Sr concentration of 1.0 ± 0.2 mg kg^−1^ dw, in *B. edulis* (one sample), and Frankowska et al. (2010) reported concentrations (*n* = 15 samples) at 0.44 ± 0.31 (0.12 to 1.3) mg kg^−1^ dw in the caps and at 0.75 ± 0.50 (0.23 to 2.1) mg kg^−1^ dw in the stems, which are within the range determined in *A. bisporus* in the present study (Table 3). Similarly, the median Sr concentrations in *X. subtomentosus* ranged from 0.10 to 0.3 mg kg^−1^ dw in the caps and from 0.19 to 1.4 mg kg^−1^ dw in the stems (Chojnacka et al. 2013).

In a series of studies on white *A. bisporus* by Vetter (1994 and 2003) and Vetter et al. (2005), Sr occurred at relatively greater concentrations, i.e. from 7.87 ± 1.25 to 9.82 ± 0.94 mg kg^−1^ dw in the caps and from 7.90 ± 1.29 to 8.96 ± 1.29 mg kg^−1^ dw in the stems (mushrooms from classical cultivation and from straw cultivation were similar in Sr content), and from 6.70 ± 0.89 to 7.47 ± 1.19 mg kg^−1^ dw in the whole fruiting bodies. Mean strontium concentrations of 0.36 ± 0.35 mg kg^−1^ dw (range from 0.04 to as high as 6.0 mg kg^−1^ dw) in white *A. bisporus* were reported in a study, relative to the quoted literature range of 4.1 to 6.7 mg kg^−1^ dw (Rzymski et al. 2017), although a lower range of 0.015 to 0.037 mg kg^−1^ dw was reported in the review by Muszyńska et al. (2017). The difference in these concentration ranges raises the question of whether the mushrooms were raised in typical (unpolluted) or polluted (made up of contaminated waste products) substrates.

The median *V* concentrations in the control *A. bisporus* mushrooms were 0.0027 mg kg^−1^ dw in the caps and 0.0062 mg kg^−1^ dw in the stems. Mushrooms grown in the Li-fortified substrate showed median values in the range from 0.0019 to 0.0039 mg kg^−1^ dw (*p* > 0.05, both M-W *U* test and Pearson) and from 0.0056 to 0.0090 mg kg^−1^ dw (*p* > 0.05, both M-W *U* test and Pearson), in the caps and stems, respectively (Table 3).

Vanadium is considered as an essential element for *A. muscaria* and also for a few other *Amanita* mushrooms, in which it usually accumulates at concentrations exceeding 100 mg kg^−1^ dw (Falandysz et al. 2007a and 2020). However, in many other mushrooms studied so far, its concentrations were low (Meisch et al. 1978), i.e. the median value was 0.11 mg kg^−1^ dw for large collections of Chanterelle *Cantharellus cibarius* as determined using ICP-DRC-MS (Falandysz et al. 2017a), or from 0.038 to 0.066 mg kg^−1^ dw (medians) in the caps of *L. scabrum* as determined using ICP-MS (Falandysz et al. 2007b).

### The elements Ag, As, Cd, Cr, Hg, Pb, Tl and U

Elements such as Ag, As, Cd, Cr, Hg, Pb, Tl and U are considered to have no physiological role in mushrooms. They are generally considered as contaminants that can be tolerated if present at ultra-trace, background, concentrations. The medians of Ag concentrations in the control *A. bisporus* were 0.07 mg kg^−1^ dw in the caps and 0.03 mg kg^−1^ dw in the stems, and mushrooms grown in the Li-fortified substrate showed higher median values in the range from 0.05 to 0.09 mg kg^−1^ dw and from 0.03 to 0.07 mg kg^−1^ dw (both at *p* < 0.05, M-W *U* test), in caps and stems, respectively (Table 3). Lithiated mushrooms, however, contained Ag at a greater concentration than the controls, but the increase was not correlated with the level of fortification (*p* > 0.05, both for caps and stems, Pearson). Silver is known to efficiently bio-concentrate in many mushroom species, usually to a greater extent in caps than stems (Borovička et al. 2010). *A. bisporus* also has high potential to bio-concentrate Ag from low-contaminated substrates (BCFs range from 50 to 120), and mushrooms cultivated using commercial substrate showed concentrations in the range of 0.15 to 0.62 mg kg^−1^ dw in the whole fruiting bodies (Falandysz et al. 1994). Some wild *Agaricus* species accumulate Ag at high concentrations, e.g. *A*. *campestris* from several locations showed cap concentrations in the range from 9.3 ± 0.3 mg kg^−1^ dw (pasture) to 62 ± 28 mg kg^−1^ dw (hippodrome), with a total reported range from 5.9 to 100 mg kg^−1^ dw (*n* = 50 caps) (Falandysz and Danisiewicz, 1995). A few *Amanita* mushrooms of the section *Lepidella* hyper-accumulate Ag in the range from 100 to 1200 mg kg^−1^ dw (Borovička et al. 2010). In comparison to the reviewed data, the Ag concentrations in *A. bisporus* in the present study can be considered as negligible (Table 3).

The median As concentrations in control *A. bisporus* were 0.17 mg kg^−1^ dw in the caps and 0.11 mg kg^−1^ dw in the stems, and mushrooms grown in the Li-fortified substrate showed median values in the range from 0.15 to 0.26 mg kg^−1^ dw (*p* > 0.05, both M-W *U* test and Pearson), and from 0.10 to 0.16 mg kg^−1^ dw (*p* > 0.05, both M-W *U* test and Pearson), in caps and stems, respectively (Table 3). Li fortification did not affect the accumulation of As which occurred at background level in the substrate.

Mushrooms usually accumulate As in small concentrations, but bio-accumulation may be higher in some species, including from the genus *Laccaria* (Falandysz and Rizal, 2016). In terms of food safety, it would be useful to identify the chemical form of As that bio-accumulates in mushrooms because inorganic forms are carcinogenic. Arsenic, as reviewed recently by Zhang et al. (2020a), occurred in the range of concentrations from 0.02 to 0.76 mg kg^−1^ dw in large collections of cultivated *A. bisporus* from farms in China*. A. bisporus* cultivated experimentally in substrate fortified with 1004 mg kg^−1^ dw of As(V) showed this metalloid in fruiting bodies at a concentration of 22.8 ± 1.0 mg kg^−1^ dw (BCF = 0.009), with a much lower concentration, 0.50 ± 0.03 mg kg^−1^ dw (BCF = 0.13), in mushrooms grown in unfortified control substrate (Soeroes et al. 2005). The As compounds found in *A. bisporus* grown in the control substrate (that was uncontaminated with the metalloid) were As(III) compounds—arsenite (< 0.008 mg kg^−1^ dw), DMA—dimethylarsinic acid (0.116 ± 0.002 mg kg^−1^ dw), MA—methylarsonic acid (0.0109 ± 0.0021 mg kg^−1^ dw), As(V)—arsenate (0.0154 ± 0.0014 mg kg^−1^ dw), AB—arsenobetaine (0.307 ± 70.07 mg kg^−1^ dw) and TMAO—trimethylarsine oxide (< 0.005 mg kg^−1^ dw). Mushrooms grown in As(V)-contaminated substrate contained high proportions of toxic As(III) and As(V) (Soeroes et al. 2005). In another cultivation experiment (Smith et al. 2007), with *A. bisporus* grown in substrate uncontaminated with As, fruiting bodies were found to bio-accumulate this element in the range from 0.34 ± 0.05 to 0.61 ± 0.5 mg kg^−1^ dw. A much greater concentration was seen in mushrooms grown in contaminated (separately with arsenic-contaminated mine waste and an arsenate solution) substrate, while the distribution of As compounds was similar to that seen in a study by Soeroes et al. (2005). The arsenic concentrations determined in lithiated and control mushrooms in the present study were at the lower end of the concentration range reported in a review that included *A. bisporus* cultivated in China (Zhang et al. 2020a), and were also lower than concentrations determined in the experiments by Soeroes et al. (2005), described above, or in a study by Smith et al. (2007).

The median Cd concentrations in the control *A. bisporus* mushrooms were 0.12 mg kg^−1^ dw in the caps and 0.08 mg kg^−1^ dw in the stems, and mushrooms grown in the Li-fortified substrate showed a similar median value range, from 0.10 to 0.22 mg kg^−1^ dw in the caps (*p* < 0.05, M-W *U* test and Pearson), but higher concentrations, i.e. from 0.09 to 0.15 mg kg^−1^ dw in the stems (*p* < 0.05, M-W *U* test) (Table 3). However, an increase of Cd in the stems did not correlate with the level of substrate fortification (*p* > 0.05, Pearson).

These values (Table 3) are within the reported range (Koyyalamudi et al. 2013) of Cd concentrations of 0.020 ± 0.021 to 0.27 ± 0.01 mg kg^−1^ dw (assuming water content at 90%) in whole commercial *A. bisporus* harvested in Australia. Lithiation of *A. bisporus* in the present study did not affect the co-accumulation of Cd. Amounts of Cd shown in the caps and stems of lithiated and control *A. bisporus* in the present study were well below the regulated limit (2.0 mg kg^−1^ dw) of this element in cultivated white *A. bisporus* in the European Union (EU1, EU 2).

The median Cr concentrations in the control *A. bisporus* were 0.009 mg kg^−1^ dw in the caps and 0.007 mg kg^−1^ dw in the stems, and mushrooms grown in the Li-fortified substrate showed median values in the range from 0.008 to 0.012 mg kg^−1^ dw in the caps (*p* > 0.05, both M-W *U* test and Pearson) and from 0.009 to 0.016 mg kg^−1^ dw in the stems (*p* > 0.05, both M-W *U* test and Pearson) (Table 3). Lithiation of *A. bisporus* in the present study did not affect the co-accumulation of Cr. The element Cr seems to be a common mineral micro-constituent of *A. bisporus*—it has been detected in the range from 1.2 ± 0.0 to 1.5 ± 0.0 mg kg^−1^ dw (assuming water content at 90%) and at 0.21 ± 0.06 (0.08 to 0.30) mg kg^−1^ dw in each batch studied by Koyyalamudi et al. (2013) and Rzymski et al. (2017).

The median Hg concentrations in the control *A. bisporus* were 0.078 mg kg^−1^ dw in the caps and 0.059 mg kg^−1^ dw in the stems, and mushrooms grown in the Li-fortified substrate showed median values in the range from 0.056 to 0.073 mg kg^−1^ dw (*p* < 0.05, M-W *U* test, and *p* > 0.05, Pearson) in the caps, and a slightly higher range from 0.041 to 0.054 mg kg^−1^ dw (*p* < 0.05, M-W *U* test, while *p* > 0.05 in the Pearson test) in the stems (Table 3).

Mercury is efficiently bio-concentrated (BCF values are around 200–300) by wild and cultivated mushrooms including *A. bisporus* (Byrne and Tušek-Žnidarič, 1990; Bressa et al. 1988; Falandysz 2017; Frank et al. 1974). Thus, the degree of Hg contamination of the substrate in which the mycelium grows is an important factor impacting the accumulation of this element in mushrooms. Genetic features of the affected species related to the availability of ligands for bio-accumulation are another contributing factor that influences the level of occurrence. Lithiation of *A. bisporus* did not affect the co-accumulation of Hg and the concentrations determined were negligible.

The median Pb concentrations in the control *A. bisporus* were 0.022 mg kg^−1^ dw in the caps and 0.035 mg kg^−1^ dw in the stems, and mushrooms grown in the Li-fortified substrate showed median values in the range from 0.015 to 0.19 mg kg^−1^ dw in the caps (*p* > 0.05, both M-W *U* test and Pearson) with higher levels, from 0.017 to 0.053 mg kg^−1^ dw, in the stems (*p* < 0.05, M-W *U* test, while *p* > 0.05, Pearson) (Table 3).

These concentrations of Pb were lower in comparison to those that have been reported for commercially produced *A. bisporus* mushrooms, which showed Pb at concentrations ranging from 0.028 to 0.148 mg kg^−1^ dw—as reviewed by Muszyńska et al. (2017). Koyyalamudi et al. (2013) reported Pb in whole *A. bisporus* in the range of 1.4 ± 0.1 to 6.2 ± 0.3 mg kg^−1^ dw (assuming water content at 90%), while an earlier study reported a mean of 0.54 ± 0.54 mg kg^−1^ dw (total range from 0.15 to 27 mg kg^−1^ dw) (Rzymski et al. 2017). The Pb content of foodstuffs is regulated within the EU, and concentrations in the caps and stems of lithiated and control *A. bisporus* in the present study were well below the maximum permitted limit of 3.0 mg kg^−1^ dw for this element, set in the EU for three species of cultivated mushroom (EU1, EU 2).

The median Tl concentrations in the control *A. bisporus* were 0.006 mg kg^−1^ dw in the caps and 0.005 mg kg^−1^ dw in the stems, and mushrooms grown in the Li-fortified substrate showed median values in the range from 0.004 to 0.006 mg kg^−1^ dw (*p* > 0.05, both M-W *U* test and Pearson) and from 0.004 to 0.006 mg kg^−1^ dw (*p* > 0.05, both M-W *U* test and Pearson), in caps and stems, respectively (Table 3).

Thallium is a toxic element accumulated in ultra-trace (< 0.1 mg kg^−1^ dw) to trace (> 0.1 mg kg^−1^ dw) levels in edible mushrooms, but there is no data on commercially cultivated white *A. bisporus*, for this element. Thallium has been determined in individual fruiting bodies of 14 mushrooms at varying concentrations, in the range from < 0.035 mg kg^−1^ dw in *Amanita rubescens* to 1.9 mg kg^−1^ dw in *Lactarius hepaticus* (Parisis and Van den Heede, 1992). The median concentration was 0.009 mg kg^−1^ dw (mean 0.015 mg kg^−1^ dw and ranged from 0.002 to 0.081 mg kg^−1^ dw) in several species of mushroom collected from forested urban areas (Pallavicini et al. 2018). The mushrooms *Suillus luteus*, *Suillus bovinus*, *B. edulis*, *Laccaria amethystina* and *Tricholoma flavovirens* showed Tl in a concentration range of 0.027 ± 0.012 to 0.10 ± 0.08 mg kg^−1^ dw, while the concentration in the wood decomposer *Armillaria solidipes* was 0.015 ± 0.003 mg kg^−1^ dw (Falandysz et al. 2001).

The median U concentrations in the control *A. bisporus* were 0.002 mg kg^−1^ dw in the caps and 0.009 mg kg^−1^ dw in the stems. Mushrooms grown in the Li-fortified substrate showed median values in the range from 0.0001 to 0.0005 mg kg^−1^ dw (*p* > 0.05, both M-W *U* test and Pearson) and from 0.0007 to 0.0011 mg kg^−1^ dw (*p* > 0.05, both M-W *U* test and Pearson), in caps and stems, respectively (Table 3).

Uranium is an element that naturally occurs in all rocks, soils and waters and comprises three alpha-emitting isotopes (^238^U, ^235^U, and ^234^U), but soils can also be contaminated with U in Technologically Enhanced Naturally Occurring Radioactive Materials (TENORM) such as industrial wastes or by-products enriched with this element (Strumińska-Parulska et al. 2020). Wild mushrooms grown in soil unpolluted with U showed concentrations in the range from 0.0011 ± 0.0005 to 0.0097 ± 0.0012 mg kg^−1^ dw (Falandysz et al. 2001). A range from non-detected (< LOD) to 0.011 mg kg^−1^ dw was noted for ectomycorrhizal mushrooms and from < LOD to 0.026 mg kg^−1^ dw in saprobic mushrooms, while unpolluted soils typically showed a concentration range that was two to three orders of magnitude greater (Borovička et al. 2011). In the present study, lithiation did not increase co-accumulation of U in *A. bisporus* and the U concentrations determined here are within the lower range of concentrations reported (Falandysz et al. 2001), in edible wild mushrooms grown in unpolluted forests.

## Conclusions

Popular white button mushrooms, *A. bisporus*, were shown to be lithiated with good yields, by cultivation in substrate fortified with Li_2_CO_3_. At the highest viable levels of substrate fortification with Li (up to 100 mg kg^−1^ dw), the fruiting bodies were enriched up to a maximum of approximately 300-fold above typical concentrations in the commercial product. Although the bio-fortification of fungi with elements such as Li is known, this study presents a highly practical, novel way of realizing this concept in a species that is well known and commonly used as a food, in a manner that could be easily adapted for wide-scale production and use. In view of published data on side effects of the Li medicinal salts, lithiated white *A. bisporus* might be considered as an alternative source of Li for pro-medicinal use. This is a promising first step, but clearly more data are needed, particularly on the next stages in using this Li food source for medicinal purposes, such as the impact of culinary or other processing on Li bioavailability from these fortified products. The Li-fortified substrate had only a slight effect on the co-accumulation of certain trace minerals such as Ag, Al, As, Ba, Cd, Co, Cr, Cs, Cu, Hg, Mn, Ni, Pb, Rb, Sr, Tl, U, V and Zn. Absolute concentrations of these elements were small and closer to the lower range of results published for commercial *A. bisporus*.

## Data Availability

Not applicable.
